# Effects of dietary pretreated Chinese herbal medicine supplementation on production performance, egg quality, uterine histopathological changes, and antioxidant capacity in late-phase laying hens

**DOI:** 10.3389/fphys.2023.1110301

**Published:** 2023-01-20

**Authors:** Ao-Chuan Yu, Min-An Wang, Li Chen, Cheng Long, Yong Guo, Xi-Hui Sheng, Xiang-Guo Wang, Kai Xing, Long-Fei Xiao, He-Min Ni, Jian-Tao Li, Xiao-Long Qi

**Affiliations:** ^1^ Animal Science and Technology College, Beijing University of Agriculture, Beijing, China; ^2^ Food Science and Engineering College, Beijing University of Agriculture, Beijing, China; ^3^ College of Animal Science and Veterinary Medicine, Shenyang Agricultural University, Shenyang, China

**Keywords:** antioxidant capacity, egg quality, late-phase laying hens, pretreated Chinese herbal medicine, performance

## Abstract

**Aims:** The study aimed to evaluate the effects of pretreated Chinese herbal medicine (PCHM) on egg quality, production performance, histopathological changes in the uterus, antiox idant capacity, and antioxidant gene expression in late-phase layers.

**Methods:** Jinghong No.1 layers (n = 360, 68 weeks old) were assigned randomly to one of f our dietary interventions. Each treatment was replicated six times. Repeat 15 chickens per g roup. All birds were fed a diet composed of a corn-soybean meal-based diet supplemented with 0, 0.2, 0.4, or 0.8% PCHM for 6 weeks.

**Results:** Dietary PCHM supplementation had no significant effects on laying rate, feed con sumption, yolk color, and shape index. With increasing PCHM level the Haugh unit linearly increased (P < 0.05). Supplementation of 0.8% PCHM increased egg weight, compared with the control (P < 0.05). PCHM can effectively alleviated the pathological changes caused by aging in the uterus including hemorrhage, and many inflammatory cell infiltrations. Supplementation of 0.4% PCHM increased glutathione peroxidase (GSHPx) in liver, magnum, and plasm considerably, compared with the control (P < 0.05). Supplementation of PCHM decr ease in the liver, magnum, and uterus on malondialdehyde (MDA) content, compared with the control (P < 0.05). Compared with the control group, mRNA expressions of glutathione peroxidase 1 (GPX1), peroxidase 4 (GPX4), catalase (CAT), and nuclear factor E2-related factor 2 (Nrf2) in the magnum, liver, and uterus were dramatically rose in the 0.4% PCHM supplementation group (P < 0.05). In summary, dietary supplementation after PCHM increased egg weight and quality in late-phase laying hens.

**Conclusion:** Dietary PCHM increased the antioxidative capacity of late-phase laying hens, which could be associated with increased mRNA expression of antioxidant enzymes and Nrf2. These findings provide potential for using PCHM to increase the production performance in late-phase laying hens.

## Introduction

The final stage of the laying cycle is characterized by a sharp drop in both egg performance (Attia et al., 2020) and egg quality (Park and Sohn, 2018). Yolk synthesis and accumulation are both reduced as egg output performance (Zakaria et al., 1983). Parallelly, egg size or weight increases, and Haugh unit (HU) and eggshell breaking strength (EBS) decrease as hens age. It has been observed that laying hens’ oviducal regression will directly affect egg production and quality (Gongruttananun et al., 2017). This may be related to the aging of oviducts. Age increases oxidant production from multiple sources, while antioxidant enzymes, the major lines of defense, decline (Zhang et al., 2015).

The activities of Chinese herbal medicines used as feed additives are extensive, including anti-apoptosis (Ibtisham et al., 2019), anti-inflammatory, and antioxidant ability (Xie et al., 2019). *Angelica* is a tonifying, nourishing, and edible herb with a long history of use, as well as for anti-inflammatory and antioxidant activity (Wei et al., 2016). Multiple live animal models of oxidative stress-related disease have shown that *Astragalus* extract offers substantial protection to the heart, kidneys, and intestines (Shahzad et al., 2016). *Epimedium* possesses multiple functions relevant in disease (Ma et al., 2011), including acting as an antioxidant (Yang et al., 2020). *Houttuynia cordata* also has medicinal properties (Wu et al., 2021), including acting as a free-radical scavenger and an anti-inflammatory (Ahn et al., 2017). Accumulating evidence has indicated that dietary herbs improve egg quality and production performance of laying hens. For example, Xie et al. (2019) found that the anti-inflammatory effect of Astragali can improve the albumen quality, and a blend of *Lonicera confusa* and *Astragali Radix* improved yolk color (Xie et al., 2019). *Epimedium* promotes follicular granulosa cell proliferation and differentiation, as well as hormone secretion and follicle development, which increases egg production rate (Guo et al., 2020a). Whole extracts or isolated compounds from *Angelica*, *Astragalus*, *Epimedium*, *Houttuynia*, and *Leonurus* can act as antioxidants. Currently, there are few studies on the five kinds of mixed Chinese herbal medicine in late-phase laying hens.

The objective of the current study was to investigate the effects of pretreated Chinese herbal medicine (PCHM) on production performance, egg quality, histopathological changes in the uterus, antioxidant capacity, and gene expression of antioxidant enzymes in late-phase laying hens.

## Materials and methods

### Animal care and use

The Beijing University of Agriculture’s Animal Care and Use Committee approved all experimental protocols (Approval ID: BUA-zc-20200073).

### Experimental materials

*Epimedium*, *Astragalus*, *Angelica*, *Leonurus*, and *Houttuynia* were all purchased from Yiren Pharmaceutical Co., Ltd. (Baoding, Hebei, China). The test bacteria and enzymolysis enzymes were purchased from Beijing challenge Biotechnology Co., Ltd. (Beijing, China). The main components of *Angelica*, *Astragalus*, *Epimedium*, *Houttuynia*, and *Leonurus* are *Angelica* polysaccharide, *Astragalus* polysaccharide, flavone, chlorogenic acid, and alkaloid, respectively (Table 1). In this experiment, *Angelica* and *Astragalus* were treated by fermentation, *Leonurus* was treated by enzymatic hydrolysis, and *Houttuynia* and *Epimedium* were not treated. According to the pre-experiment, the main components of *Angelica* and *Astragalus* reach their highest level through fermentation, the main components of *Leonurus* through enzymatic hydrolysis, and the main components of *Houttuynia* and *Epimedium* through no treatment at all. According to the Chinese veterinary medicine code, the mixing ratio of *Epimedium*, *Astragalus*, *Angelica*, *Leonurus*, and *Houttuynia* after pretreatment is 1:2:2:1:2. After processing, the mixed herbs were called PCHM. Fermentation conditions: *Bacillus licheniformis*, *Bacillus coagulans*, *Aspergillus niger*, *Bacillus subtilis* (1:1:1:1), conditions: time: 5 days, temperature: 37°C, water content: 45%. Enzymatic hydrolysis conditions: complex enzyme (pectinase, cellulase, laccase), conditions: time: 4 h, temperature: 55°C, water content: 45%.

**TABLE 1:** Main ingredients of the pretreated Chinese herbal medicine.

Item	Control group
Angelica	Angelica polysaccharide
Astragalus	Astragalus polysaccharide
Epimedium	Flavone
Houttuynia	Chlorogenic acid
Motherwort	Alkaloid

### Birds and diets

A total of three hundred and sixty 68-week-old Jinghong No. 1 laying hens (initial body weights 1.65 ± 0.10 kg) with a similar weight and genetic background were used. A completely randomized design was used to divide the birds into the four treatment groups. Each treatment had six replicates with 15 birds each. All birds were fed a basal diet for 1 week before being assigned a diet containing maize-soybean meal containing 0, 0.2, 0.4, or 0.8% PCHM for 6 weeks. Table 2 displays the composition and nutritional levels of the diet based on maize-soybean meal. The experimental diet feeding period lasts for 6 weeks and the birds in the experiment were exposed to 16-h light cycles and had free access to water and the experimental diets at all times.

**TABLE 2:** The composition and nutritional level of the basic diet (air-dried basis).

Ingredients	Content (%)	Nutrient level	Content (%)
Corn	62.0	Metabolizable energy, MJ·kg^−1^	11.2
Soybean meal	24.0	Crude protein (%)	16.6
Soybean oil	1.00	Calcium (%)	3.26
Limestone	8.00	Available phosphorus (%)	0.40
Dicalcium phosphate	1.80	dl-Methionine (%)	0.34
Salt	0.35	l-Lysine (%)	0.82
dl-Methionine	0.10		
Premix^a^	2.75		
Total	100		

^a^ The premix provided the following per kilogram of the diet: VA 12,500 IU, VD3 5,250 IU, VE 21.25 mg, VK3 4.375 mg, VB1 2.5 mg, VB2 11.25 mg, VB6 6.25 mg, VB12 3 mg, nicotinic acid 50 mg, D-pantothenic acid 40.75 mg, folic acid 6 mg, biotin 2.375 mg, Fe 87.5 mg, Zn 68 mg, Cu 9.5 mg, Mn 75 mg, I 1.5 mg, and Se 0.3 mg.

### Sample collection

Every day, data on egg production and weight were logged. The number of eggs produced was expressed as an average hen-day production, which was derived by dividing the total number of hen-days by the number of eggs. Average egg weight was calculated as total egg weight divided by the number of eggs. Feed consumption was recorded on a replicate basis at weekly intervals. The feed conversion ratio was measured as the amount of feed consumed relative to the amount of eggs produced in kilograms. Egg quality was measured on three eggs collected randomly from each replicate on the 14th, 28th, and 42nd days. One healthy bird was chosen at random from each replicate at the end of the feeding period (one bird per replicate, and 24 birds in total). Exsanguination of the left jugular vein with scalpels was used to collect blood samples, which were then centrifuged at 4°C at 4,000 g for 10 min to separate plasma. Following collection, plasma samples were flash-frozen at −80°C and stored in the freezer until analysis. Birds were euthanized by exsanguination and necropsied, and the liver, magnum, and uterus were separated immediately and quickly frozen at −80°C for further analysis. Approximately 2 cm medial portion sections of the uterus were removed and cleaned thoroughly with 0.9% saline, then placed in 4% formaldehyde solution for tissue fixation and histological measurement.

### Egg quality and performance measurement

The rate of egg production was determined as follows: egg production rate = eggs laid per day/(birds counted per day). Feed efficiency was calculated weekly. Haugh units, yolk color, and albumen height were measured using an egg analyzer (Orka Food Technology Ltd., Ramat Hasharon, Israel). Yolk color was defined according to the Roche yolk color fan, where 1 represents bright yellow and 15 represents dark yellow. An egg force reader (Herzliya, Tel Aviv, Israel) was used to measure eggshell strength. The thickness of the eggshell (ST) was calculated as follows: [ST, mm = SW/(ES d)], where SW is the weight of the eggshell, ES is the surface area of the egg, and d is the density of the material (2.3 g/cm^3^ for calcium carbonate).

### Histopathologic evaluation of uterine tissue

Uterine tissues were fixed in 4% paraformaldehyde and embedded in paraffin, and the 5 µm-thick sections were stained with hematoxylin-eosin (H&E). Samples were trimmed, dehydrated, embedded, sliced, dyed, and sealed in strict accordance with the standard operating procedures for pathological experiment detection of the service-bio unit (Liu et al., 2022). Histological samples were evaluated for degree of injury, using variables such as the integrity of tissue structure and the numbers of infiltrated inflammatory cells (service-bio, Wuhan, China).

### Measurement of antioxidant enzyme activity

The plasma, liver, magnum, and uterus tissue concentrations of glutathione peroxidases (GSH-Px), catalase (CAT), superoxide dismutase (SOD), and the ability to scavenge superoxide anion radicals and the hydroxyl radical malondialdehyde (MDA) were measured by enzyme-linked immunosorbent assay (ELISA) using a commercial ELISA kit for chicken (Nanjing Jiancheng Bioengineering Institute, Nanjing, China; catalog no. A003-1-2, A007-1-1, A001-1-2, A005-1-2, A052-1-1, A018-1-1) according to the manufacturer’s instructions. T-SOD activity was determined by using the xanthine/xanthine oxidase method, which is based on the inhibition of nitroblue tetrazolium formazan. The activity of GSH-Px was measured using H_2_O_2_ as a substrate in the presence of reduced glutathione. The GSH-Px activity was reported in micromoles of oxidized NADPH per minute.

### Quantitative PCR analysis

Total RNA was extracted from 0.1 g of liver, magnum, and uterus tissue sample using TRIzol reagent (Thermo Fisher Scientific, Shanghai, China), as per the manufacturer’s instructions, and then reverse-transcribed into single-stranded cDNA using the Thermo First cDNA Synthesis Kit (Promega, Beijing, China). The expressions of nuclear factor E2 related factor 2 (*Nrf2*), Cu-Zn superoxide dismutase (*SOD*) *1*, Mn superoxide dismutase (*SOD*) *2*, catalase (*CAT*), glutathione peroxidase 1 (*GPX1*), and peroxidase 4 (*PX4*) were determined using qRT-PCR with specific primers (Table 3). An AriaMx Real-Time PCR system (Agilent Technologies, Santa Clara, California, United States) was used for quantitative PCR analysis. After initial denaturation at 95°C for 10 min, 40 cycles of amplification were carried out (95°C for 10 s and 58.2°C for 30 s), followed by the generation of melt curves that could be used to verify the specificity of amplification. The samples were tested in triplicate. The 2^−ΔΔCT^ (Livak and Schmittgen, 2001) method was used to calculate the relative gene expression levels. Refer to the operation of (Madkour et al., 2021) for specific process.

**TABLE 3:** Primer sequences used for quantitative real-time PCR.

Gene	Primer sequence (5′-3′)	Fragment size (bp)	Accession number
*CAT*	Forward: ACC​AAG​TAC​TGC​AAG​GCG​AAA​GT Reverse: ACC​CAG​ATT​CTC​CAG​CAA​CAG​TG	91	NM_001031215.2
*SOD1*	Forward: TTG​TCT​GAT​GGA​GAT​CAT​GGC​TTC Reverse: TGC​TTG​CCT​TCA​GGA​TTA​AAG​TGA​G	98	NM_205064
*SOD2*	Forward: CAG​ATA​GCA​GCC​TGT​GCA​AAT​CA Reverse: GCA​TGT​TCC​CAT​ACA​TCG​ATT​CC	86	NM_204211.1
*GPX1*	Forward: TTC​GAG​AAG​TTC​CTC​GTG​GG Reverse: CCT​GCA​GTT​TGA​TGG​TCT​CG	79	NM_0012778553.2
*GPX4*	Forward: TCA​ACC​GTG​AGG​GCC​AAG​T Reverse: CTCGGCACGCAGCTCTAC	100	NM_001346448.1
*Nrf2*	Forward: ACA​TGG​ACA​GTT​CTC​CTG​GG Reverse: CGG​CTC​CAC​AGA​AGG​AAG​TA	92	NM_205117.1
*β-Actin*	Forward: GCC​AAC​AGA​GAG​AAG​ATG​ACA​C Reverse: GTA​ACA​CCA​TCA​CCA​GAG​TCC​A	118	NM_205518

### Statistical analysis

All statistical analyses were performed using SPSS 22.0 (IBM Corp., Armonk, NY, United States). Data are presented as mean ± standard deviation of the mean (SD). Additionally, polynomial regression analysis was used to test the linear and quadratic nature of the response to the additive PCHM dosage, and Tukey’s multiple comparison tests were used to analyze the differences among various treatments. *p* < 0.05 was considered significant.

## Results

### Comparison of components of Chinese herbal medicine

Comparison of the main components of five Chinese herbal medicines is shown in Table 4. *Angelica*, *Astragalus* fermentation treatment, *Leonurus* enzymolysis treatment, *Houttuynia*, and *Epimedium* did not have treatment.

**TABLE 4:** Comparison of different treatment methods of Chinese herbal.

Item	Control group	Enzymolysis	Fermentation	SEM	*p*-value		
					ANOVA	Linear	Quadratic
*Angelica* (%)	7.74^b^	8.28^b^	17.1^a^	0.29	<0.01	0.07	0.94
*Astragalus* (%)	10.6^b^	11.9^b^	21.1^a^	0.14	<0.01	<0.01	0.03
*Epimedium* (%)	7.58	7.99	8.14	0.21	0.21	0.23	0.94
*Houttuynia* (%)	11.9	11.6	12.8	0.01	0.40	0.33	0.33
*Leonurus* (%)	1.39^b^	2.52^a^	1.37^b^	0.08	<0.01	<0.01	0.09

^a–b^ Means within a row with no common superscripts differ significantly (P<0.05).

### Performance and egg quality

The effects of dietary supplementation of PCHM on performance and egg quality are shown in Table 5 and Table 6. PCHM supplementation had no effect on laying rate, feed consumption, yolk color, shell thickness, shape index; PCHM supplementation had a significant effect on feed efficiency, egg weight, albumen height, and eggshell strength (*p* < 0.05). With increasing dietary supplementation levels of PCHM, the Haugh unit increased linearly (*p* < 0.05).

**TABLE 5:** Effect of dietary PCHM supplementation on performance.

Item	Time (week)	PCHM (%)				SEM	*p*-value		
		0	0.2	0.4	0.8		ANOVA	Linear	Quadratic
Laying rate (%)	1–2	82.4	81.4	85.3	85.5	0.95	0.32	0.13	0.76
	3–4	80.3	82.3	85.2	83.4	0.89	0.10	0.11	0.05
	5–6	79.5	84.6	85.1	83.5	0.95	0.35	0.16	0.33
	1–6	80.7	82.8	85.2	84.1	0.68	0.11	<0.05	0.24
Feed efficiency (kg/kg)	1–2	2.38	2.35	2.29	2.25	0.03	0.52	0.14	0.96
	3–4	2.49^a^	2.29^b^	2.36^b^	2.28^b^	0.03	<0.05	<0.05	0.20
	5–6	2.48	2.34	2.31	2.26	0.03	0.07	<0.05	0.46
	1–6	2.45^a^	2.33^a^	2.32^a^	2.27^b^	0.02	<0.05	<0.01	0.43
Feed consumption (g/d)	1–2	120	120	120	120	0.17	0.87	0.47	0.76
	3–4	121	121	121	121	0.26	0.91	0.71	0.91
	5–6	122	122	121	121	0.20	0.19	<0.05	0.76
	1–6	121	121	121	121	0.14	0.87	0.42	0.94
Egg weight (g/egg)	1–2	61.2	62.2	61.4	62.5	0.28	0.28	0.23	0.94
	3–4	61.3^b^	63.6^a^	61.7^b^	63.2^a^	0.25	<0.01	0.07	0.94
	5–6	61.5	63.2	61.6	63.2	0.35	<0.05	0.13	0.55
	1–6	61.3^b^	63.0^a^	61.5^b^	63.0^a^	0.23	<0.01	<0.05	0.75

^a–d^ Means within a row with no common superscripts differ significantly (*p* < 0.05).

**TABLE 6:** Effect of dietary PCHM supplementation on egg quality.

Item	Time (week)	PCHM (%)				SEM	*p*-value		
		0	0.2	0.4	0.8		ANOVA	Linear	Quadratic
Haugh unit	1–2	66.7	68.7	66.2	69.3	0.90	0.59	0.54	0.78
	3–4	66.2^b^	66.3^b^	68.4^ab^	73.5^a^	1.02	<0.05	<0.01	0.17
	5–6	70.4	72.6	73.9	75.4	1.01	0.35	0.08	0.87
	1–6	67.8^b^	69.2^ab^	69.5^ab^	72.7^a^	0.59	<0.05	<0.01	0.37
Yolk color	1–2	5.11	6.11	5.94	5.94	0.15	0.05	0.06	0.07
	3–4	5.55	5.95	5.53	5.58	0.09	0.32	0.68	0.34
	5–6	6.17	6.44	6.59	6.83	0.13	0.24	<0.05	0.99
	1–6	5.61	6.14	6.04	6.12	0.09	0.09	0.06	0.17
Albumen height	1–2	5.08	5.13	4.92	5.25	0.09	0.68	0.71	0.48
	3–4	4.81^b^	4.63^b^	4.77^b^	5.69^a^	0.12	<0.01	<0.01	0.03
	5–6	5.71^b^	5.84^ab^	5.89^ab^	6.45^a^	0.12	<0.01	<0.05	0.34
	1–6	5.08^b^	5.22^b^	5.17^b^	5.78^a^	0.08	<0.01	<0.01	0.09
Eggshell strength (N/cm^2^)	1–2	2.49^b^	2.96^a^	2.88^a^	2.82^ab^	0.06	0.01	0.04	<0.01
	3–4	2.62	3.0	3.17	2.74	0.10	0.15	0.60	<0.05
	5–6	2.91	2.96	2.96	2.94	0.08	0.99	0.90	0.83
	1–6	2.67	3.00	3.00	2.83	0.05	0.06	0.26	<0.05
Shell thickness (μm)	1–2	0.35	0.35	0.35	0.35	0.01	0.17	0.78	<0.05
	3–4	0.35	0.35	0.35	0.35	0.01	0.17	0.78	<0.05
	5–6	0.35	0.35	0.35	0.34	0.01	0.50	0.33	0.33
	1–6	0.35	0.35	0.35	0.35	0.00	0.20	0.93	<0.05
Shape index	1–2	1.31	1.30	1.32	1.30	0.01	0.93	0.98	0.80
	3–4	1.31	1.33	1.31	1.32	0.01	0.92	0.86	0.96
	5–6	1.32	1.32	1.33	1.32	0.01	0.96	0.88	0.95
	1–6	1.32	1.31	1.31	1.32	0.04	0.99	0.96	0.87

^a–d^ Means within a row with no common superscripts differ significantly (*p* < 0.05).

### Histopathological analysis

**FIGURE 1:**
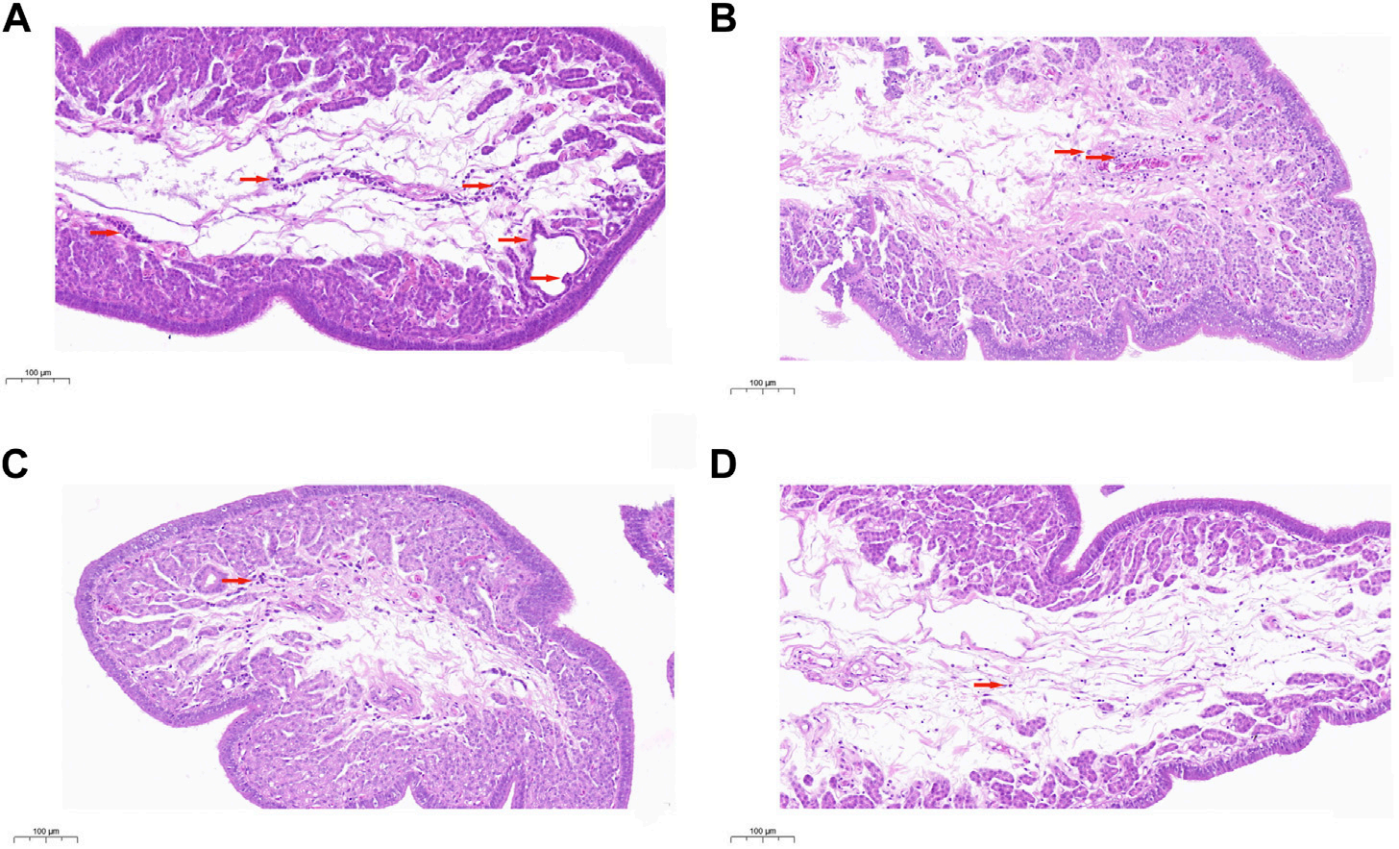
Tissues from the uterus were removed and stained with H&E. Scale bar: 100 μm. **(A)** Uterine histopathology in the control group; **(B–D)** Uterine histopathology with PCHM treatment groups at 0%, 0.2%, 0.4%, and 0.8%, respectively. The locations of tissue damage and inflammatory cell infiltration are denoted by the directional arrows in red.

The histological analysis was performed to assess the pathological changes in uterine tissues. The results demonstrated that aging resulted in severe injury, including hyperemia, hemorrhage, and many inflammatory cell infiltrations (Figure 1A). However, the aging-induced pathological changes were dramatically improved by PCHM at doses of 0.2 (Figure 1B), 0.4 (Figure 1C), and 0.8% (Figure 1D). These results indicate that PCHM could effectively attenuate aging-induced pathological changes.

### Antioxidant enzyme activity and free-radical scavenging ability

**FIGURE 2:**
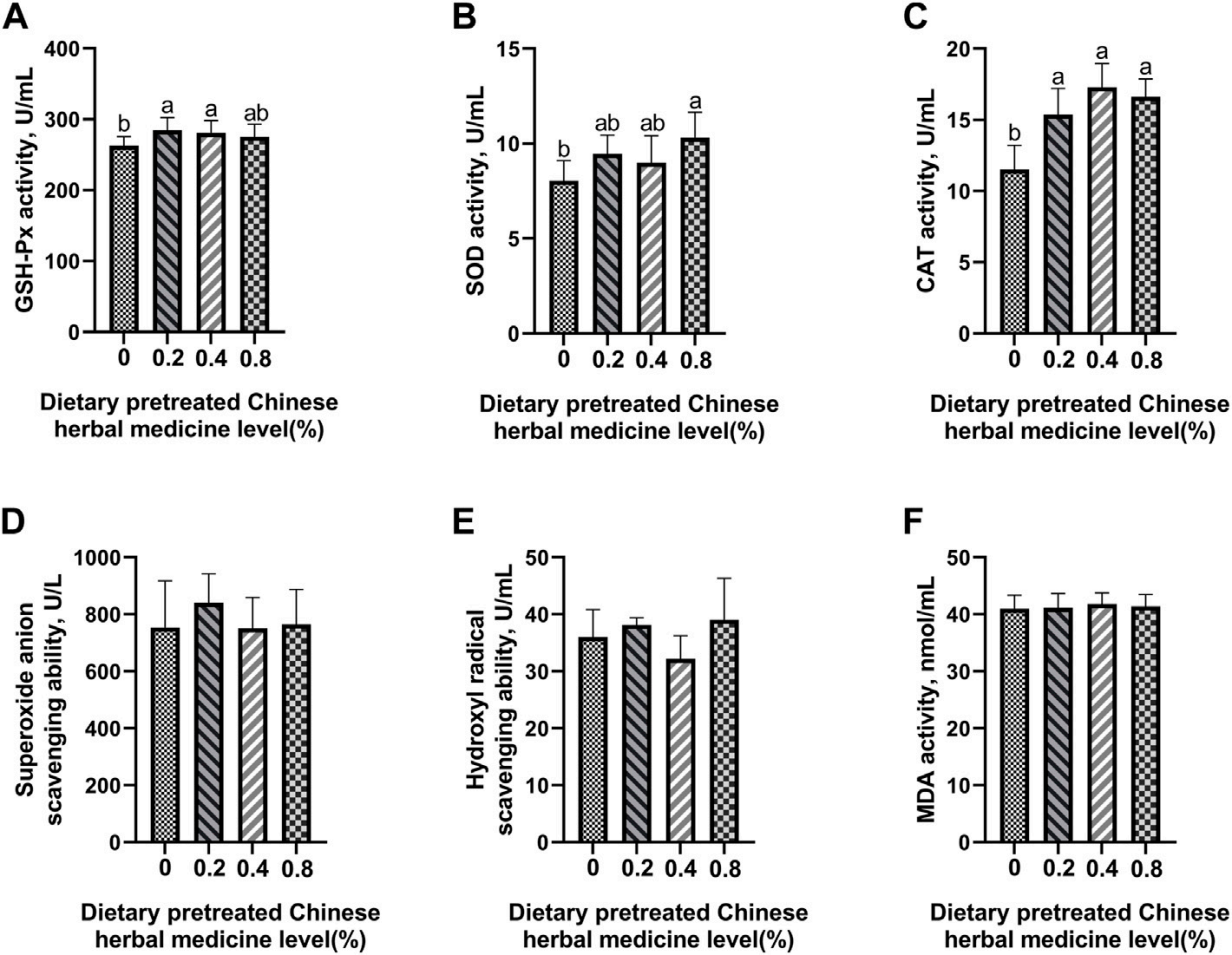
Effect of dietary PCHM supplementation on the antioxidant enzyme activity and free radicals in the plasma of late-phase laying hens. **(A–C)** Glutathione peroxidase (GSH-Px), superoxide dismutase (SOD), and catalase (CAT) activity in the plasma. **(D,E)** Scavenging free radical abilities in the plasma. **(F)** Malondialdehyde (MDA) level in the plasma. Values are expressed as means ± SEM of six birds per treatment. Means without a common letter differ (*p* < 0.05).

**FIGURE 3:**
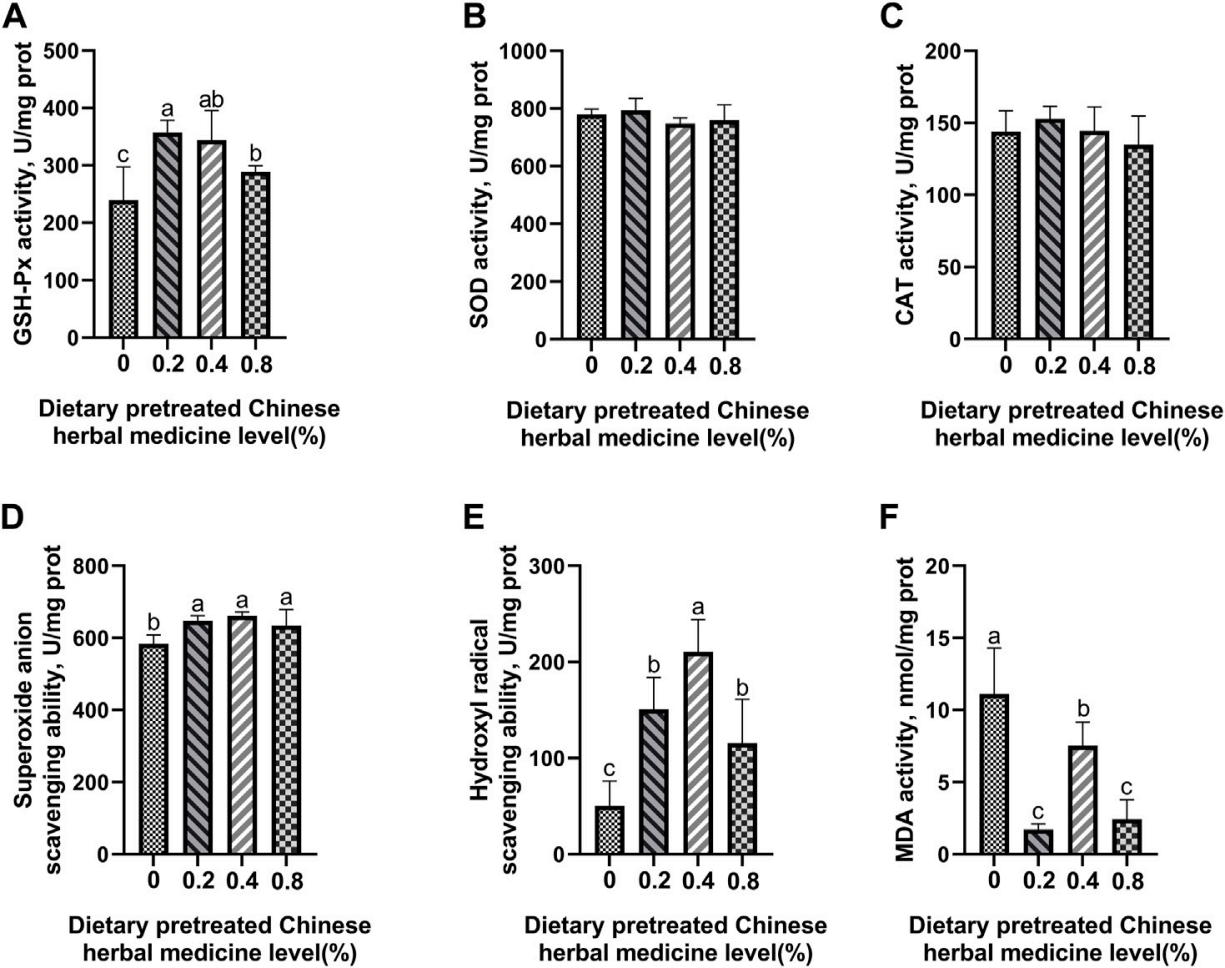
Effect of dietary PCHM supplementation on the antioxidant enzyme activity and free radicals in the liver of late-phase laying hens. **(A–C)** Glutathione peroxidase (GSH-Px), superoxide dismutase (SOD), and catalase (CAT) activity in the liver. **(D,E)** Scavenging free radical abilities in the liver. **(F)** Malondialdehyde (MDA) level in the liver. Values are expressed as means ± SEM of six birds per treatment. Means without a common letter differ (*p* < 0.05).

**FIGURE 4:**
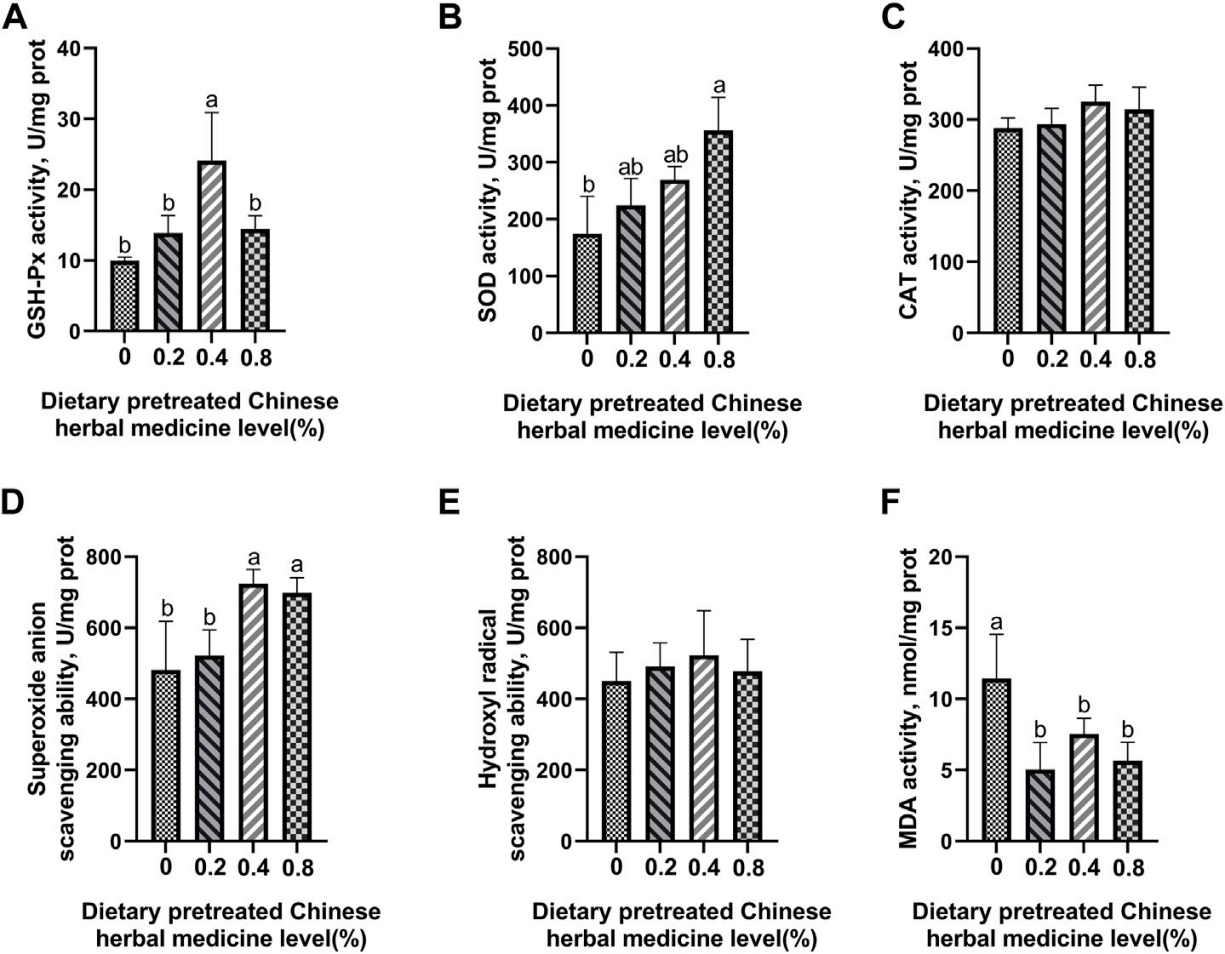
Effect of dietary PCHM supplementation on the antioxidant enzyme activity and free radicals in the magnum of late-phase laying hens. **(A–C)** Glutathione peroxidase (GSH-Px), superoxide dismutase (SOD), and catalase (CAT) activity in the magnum. **(D,E)** Scavenging free radical abilities in the magnum. **(F)** Malondialdehyde (MDA) level in the magnum. Values are expressed as means ± SEM of six birds per treatment. Means without a common letter differ (*p* < 0.05).

**FIGURE 5:**
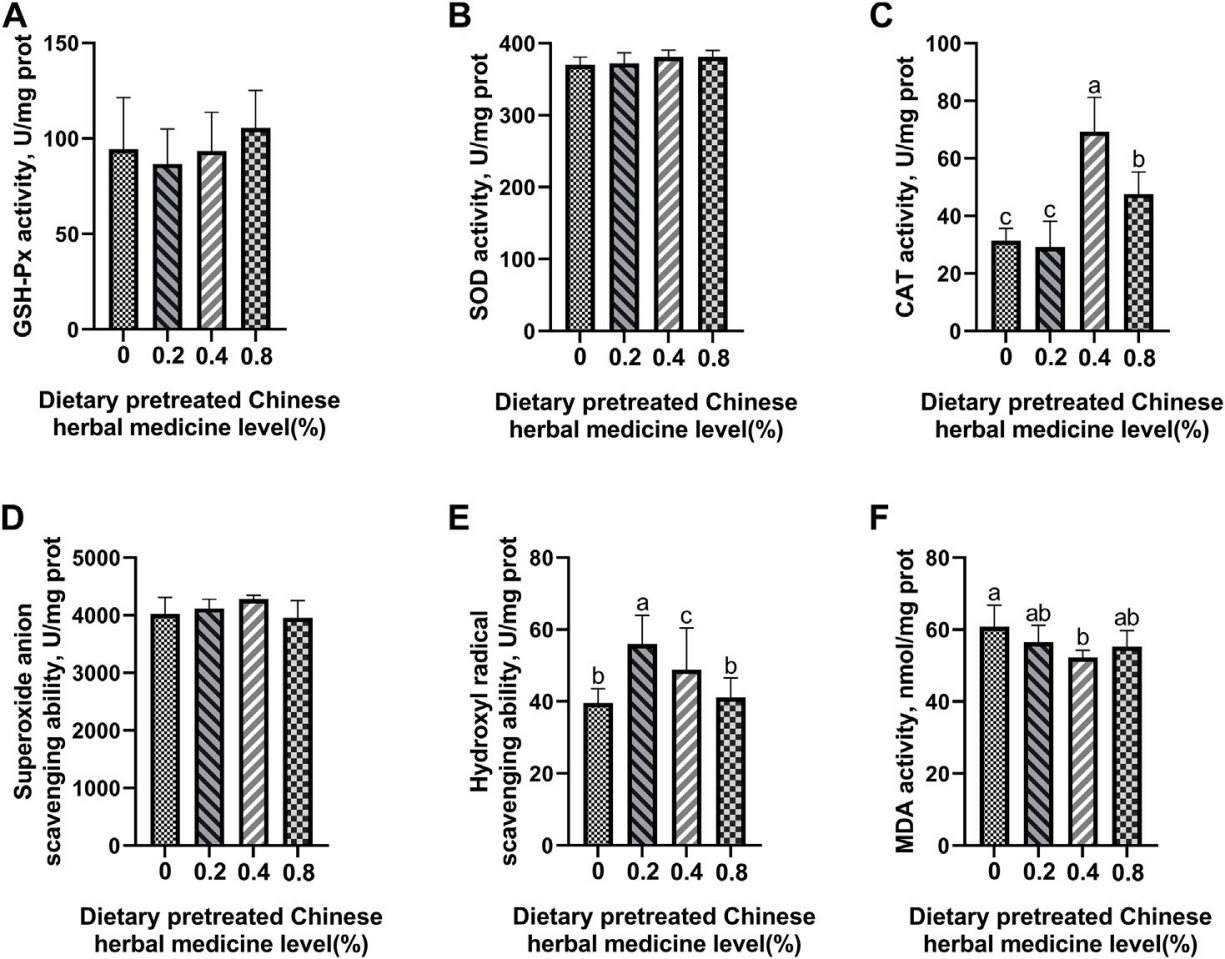
Effect of dietary PCHM supplementation on the antioxidant enzyme activity and free radicals in the uterus of late-phase laying hens. **(A–C)** Glutathione peroxidase (GSH-Px), superoxide dismutase (SOD), and catalase (CAT) activity in the uterus*.* **(D,E)** Scavenging free radical abilities in the uterus. **(F)** Malondialdehyde (MDA) level in the uterus. Values are expressed as means ± SEM of six birds per treatment. Means without a common letter differ (*p* < 0.05).

The effect of dietary PCHM supplementation on the antioxidant enzyme activity and free radical scavenging capabilities in plasma, liver, magnum, and uterus are depicted in Figures 2–5, respectively. The PCHM supplementation significantly influenced the antioxidant enzyme activity and free radical scavenging capabilities in plasma, liver, magnum, and uterus. In the plasma, a significant (*p* < 0.05) increased in the protein level of CAT and GSH-Px was noticed in the 0.2% and 0.4% PCHM groups, and CAT and SOD in 0.8% PCHM group supplemented were observed as compared to the other experimental groups. In liver, a significant (*p* < 0.05) increased in the protein level of GSH-Px and free radical scavenging capabilities was noticed in the PCHM supplementation as compared to the control. Further, in liver a significant (*p* < 0.05) decreased the content of MDA in the PCHM supplementation as compared to the control. In magnum, a significant (*p* < 0.05) increased of GSH-Px protein level in the 0.4% PCHM groups, SOD protein level in 0.8% PCHM group, the ability to scavenge superoxide anion in 0.4% and 0.8% PCHM groups were observed as compared to the other experimental groups. Further, in magnum a significant (*p* < 0.05) decreased the content of MDA in the PCHM supplementation as compared to the control. Similarly, in uterus, a significant (*p* < 0.05) increased of CAT protein level in the 0.4% and 0.8% PCHM groups, the ability to scavenge hydroxyl radical in 0.2% and 0.4% PCHM group were observed as compared to the other experimental groups. Further, in uterus, significantly (*p* < 0.05) decreased the content of MDA in the 0.4% PCHM groups were evident as compared to the other experimental groups.

### Antioxidant enzymes and Nrf2 mRNA expression

**FIGURE 6:**
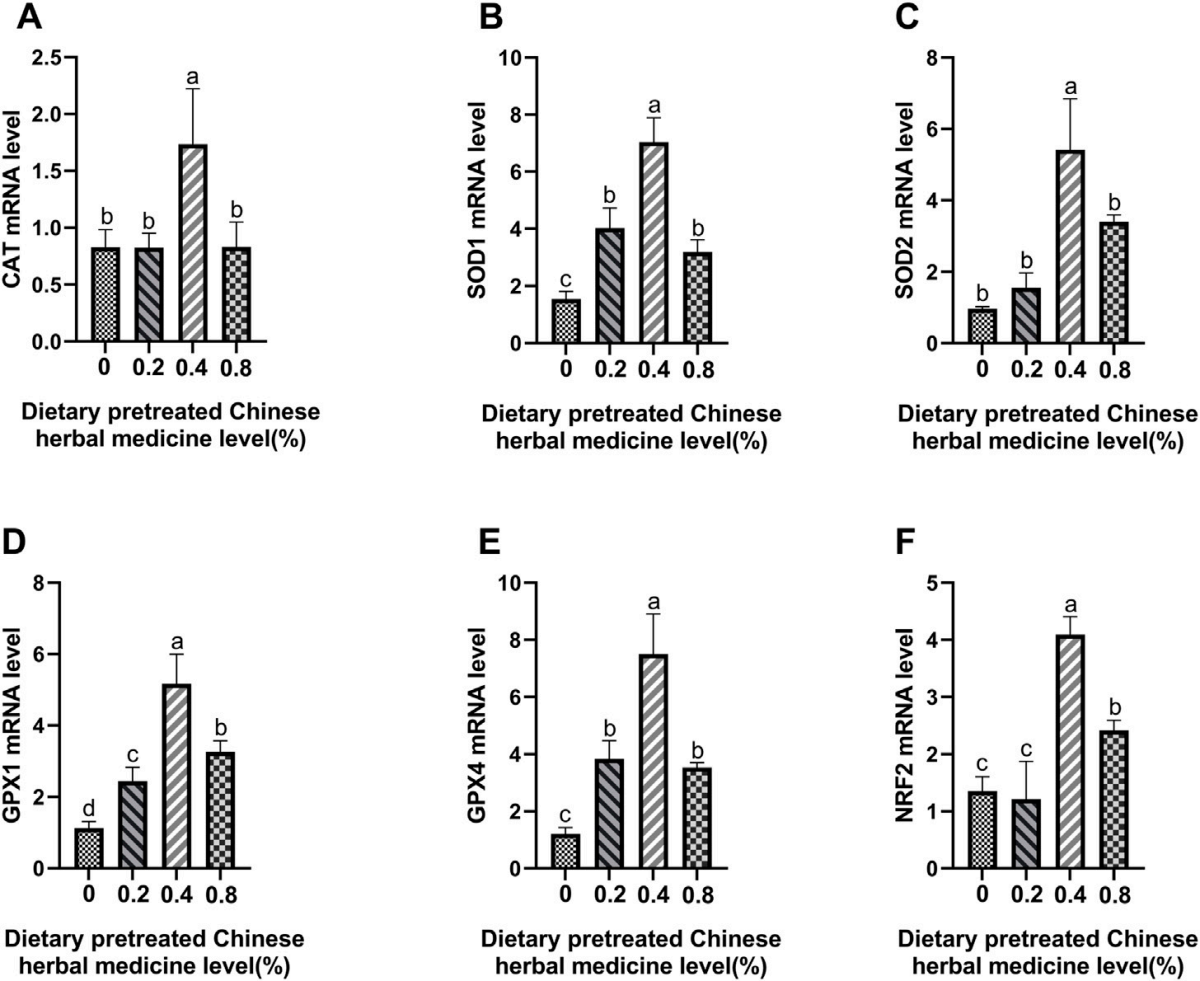
Effects of dietary PCHM supplementation on antioxidation-related mRNA expression in liver. The effect of adding PCHM (0, 0.2%, 0.4%, 0.8%) in diets in the liver, measured by quantitative PCR: **(A)** *CAT*, **(B)** *SOD1*, **(C)** *SOD2*, **(D)** *GPX1*, **(E)** *GPX4*, and **(F)** *Nrf2*. Values are expressed as means ± SEM of six birds per treatment. Means without a common letter differ (*p* < 0.05).

**FIGURE 7:**
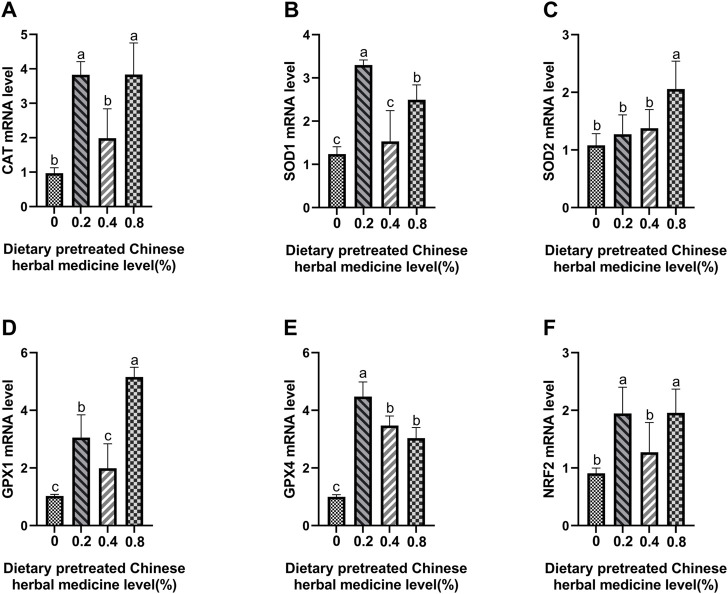
Effects of dietary PCHM supplementation on antioxidation-related mRNA expression in magnum. The effect of adding natural herbs (0, 0.2%, 0.4%, 0.8%) in diets in the magnum, measured by quantitative PCR: **(A)** *CAT*, **(B)** *SOD1*, **(C)** *SOD2*, **(D)** *GPX1*, **(E)** *GPX4*, and **(F)** *Nrf2*. Values are expressed as means ± SEM of six birds per treatment. Means without a common letter differ (*p* < 0.05).

**FIGURE 8:**
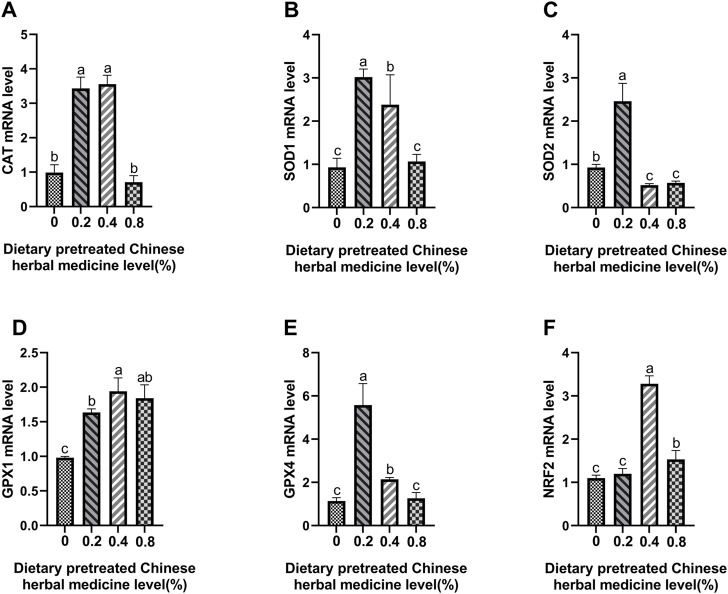
Effects of dietary PCHM supplementation on antioxidation-related mRNA expression in uterus. The effect of adding PCHM (0, 0.2%, 0.4%, 0.8%) in diets in the uterus, measured by quantitative PCR: **(A)** *CAT*, **(B)** *SOD1*, **(C)** *SOD2*, **(D)** *GPX1*, **(E)** *GPX4*, and **(F)** *Nrf2*. Values are expressed as means ± SEM of six birds per treatment. Means without a common letter differ (*p* < 0.05).

The effect of dietary PCHM supplementation on the expressions of *CAT*, *SOD1*, *SOD2*, *GPX1*, *GPX4*, and *Nrf2* genes in liver, magnum and uterus are depicted in Figures 6–8, respectively. The PCHM supplementation significantly influenced the expression of these genes in liver, magnum, and uterus. Expression of all the genes was significantly (*p* < 0.05) upregulated in liver with 0.4% PCHM supplementation as compared to the control. In magnum, a significant (*p* < 0.05) increase in the expression of *CAT* and *Nrf2* was noticed in the 0.2% and 0.8% PCHM supplemented as compared to the other groups. Further, in magnum, significantly (*p* < 0.05) greater expression of *SOD1* and *GPX4* in the 0.2 PCHM groups, and *SOD2* and *GPX1* in the 0.8% PCHM group were evident as compared to the other experimental groups. Similarly, in uterus, a significant (*p* < 0.05) upregulated expression of *CAT*, *SOD1* and *GPX4* in the 0.2% and 0.4% PCHM groups, *SOD2* in 0.2% PCHM group, *GPX1* in 0.2%, 0.4%, and 0.8% PCHM groups, and *Nrf2* in the 0.4% and 0.8% PCHM groups were observed as compared to the other experimental groups.

## Discussion

The purpose of this study was to investigate how dietary supplementation of PCHM affected production performance, egg quality, histopathological changes in the uterus, antioxidant capacity, and antioxidant enzyme gene expression in late-phase laying hens. Egg quality is important in commercial layers (Odabasi et al., 2007). The quality of eggs, notably their shells, gradually decreases as hens age past their prime laying years (Bain et al., 2016). It has been reported that supplementation of laying hen diets with herbs can improve their egg-laying rate (Li et al., 2017), Haugh unit (Moon et al., 2021), and shell strength (Xiao et al., 2019). The present study revealed that dietary supplementation of PCHM increased average egg weight, shell thickness, eggshell strength, Haugh unit and albumen height, but did not affect the laying rate, feed consumption, yolk color, or shape index. With increasing dietary levels of PCHM, the Haugh unit increased linearly. The improvement of Haugh unit may be caused by the polysaccharide contained in PCHM (Guo et al., 2020b). Similar findings were reported by Xie et al. (2019), who observed that diets including herbal medicine (*Lonicera confusa* and *Astragali Radix*) improved the albumen quality in laying hens. The antioxidant ability of PCHM’s bioactive components is responsible for these changes in Haugh unit. In particular, prior investigations (Costa et al., 2019) have revealed the antioxidant capabilities of PCHM, which likely prevent protein oxidation in eggs.

To explore a possible mechanism for the improvement in egg qualities, and feed efficiency in late-phase laying hens with PCHM supplementation, histopathological changes in the uterus and antioxidant status were determined. The ability to ovulate was closely related to the uterus (Kong et al., 2012). Aging causes certain changes in the tissue morphology of uterus such as swelling of collagen fibres, elastic tissue fragments and oedema (Mulholland and Jones, 1993). Bitter ginseng bases extracted from the Chinese herb bitter ginseng were reported to significantly reduce uterine damage in a mouse model induced by *Staphylococcus aureus* lipophosphatidic acid (Jiang et al., 2019). In the current study, PCHM supplementation attenuated the uterine injury in late-phase laying hens, which the improvement of uterine injury was probably related to the ability of PCHM to inhibit lipid peroxidation (Sikiru et al., 2019). Recent research too demonstrates that ginsenoside ameliorates pathological uterine damage by boosting the anti-oxidant enzymes SOD and GSH (He et al., 2021).

Oxidative stress contributes to aging and age-related disorders (Miyazawa et al., 2009). MDA is a lipid peroxidation marker used to evaluate lipid peroxidation as a result of elevated oxidative stress (Ottolenghi et al., 2019). Oxidative stress may come from either an excess of free radical generation or a breakdown of antioxidant defense systems (Torun et al., 2009). It has been reported that some herbs possess strong radical-scavenging ability (Jarco et al., 2021). In the current study, PCHM was added to the diet to effectively improve the free-radical scavenging capacity and reduce the content of MDA in liver, magnum, and uterus. A previous report indicates that supplementation of 0.8 g/kg herbs lowers MDA content in egg yolk by increasing antioxidant enzyme activity (Chen et al., 2018). Recent studies have shown that improvement of free-radical scavenging ability may be related in part to antioxidant enzyme activity (Heng et al., 2021). It is reported that GSH-Px is an important antioxidant enzyme that can convert hydrogen peroxide into water (Zhou et al., 2021). Similarly, dietary supplementation of ginger powder increases the antioxidant enzymatic activity of laying hens (Lee et al., 2012). These results of the current study indicate that dietary supplementation with PCHM at 0.4% was most effective in improving the antioxidant capacity of late-phase laying hens.

Superoxide dismutases (SODs) are an important class of metallo-antioxidant enzymes in the metabolism of ROS in living organisms (Zhao et al., 2021). Selenium (Se) is of great importance in the treatment of diseases caused by oxidative stress (Rashid and Coombs, 2019). Previous studies have shown that selenomethionine promotes the expression of *Nrf2* transcription factor-related genes in cells during lipopolysaccharide stimulation (Adeniran et al., 2022). The change in antioxidant enzyme activity is related to gene expression (Guo et al., 2014). Previous research has demonstrated that the addition of tanshinone can boost *GPX1* mRNA expression in macrophages (Li et al., 2008). Dietary supplementation with PCHM elevated the mRNA expression of *CAT*, *SOD1*, *SOD2*, *GPX1*, and *GPX4* in the liver and uterine in the current study. In particular, dietary supplementation with 0.4% PCHM is most effective in the liver.

Nrf2 is regarded as an important regulator of the cellular antioxidant response (Motohashi and Yamamoto, 2004). Studies have shown that with the overexpression of *Nrf2*, its downstream gene expression levels were significantly upregulated (Xiao et al., 2020; Xue et al., 2021). *Nrf2*-null affects mRNA expression of *SOD1*, *SOD2*, and *CAT* (Reisman et al., 2009). A previous study showed that theaflavin promotes resistance to oxidative stress-induced cell damage by activating *Nrf2* (Li et al., 2021). In this research, adding PCHM to the diet dramatically increased *Nrf2* mRNA levels in the liver and the uterine. The reason for improvement is related to the polysaccharide contained in PCHM (Liu et al., 2022).

**FIGURE 9:**
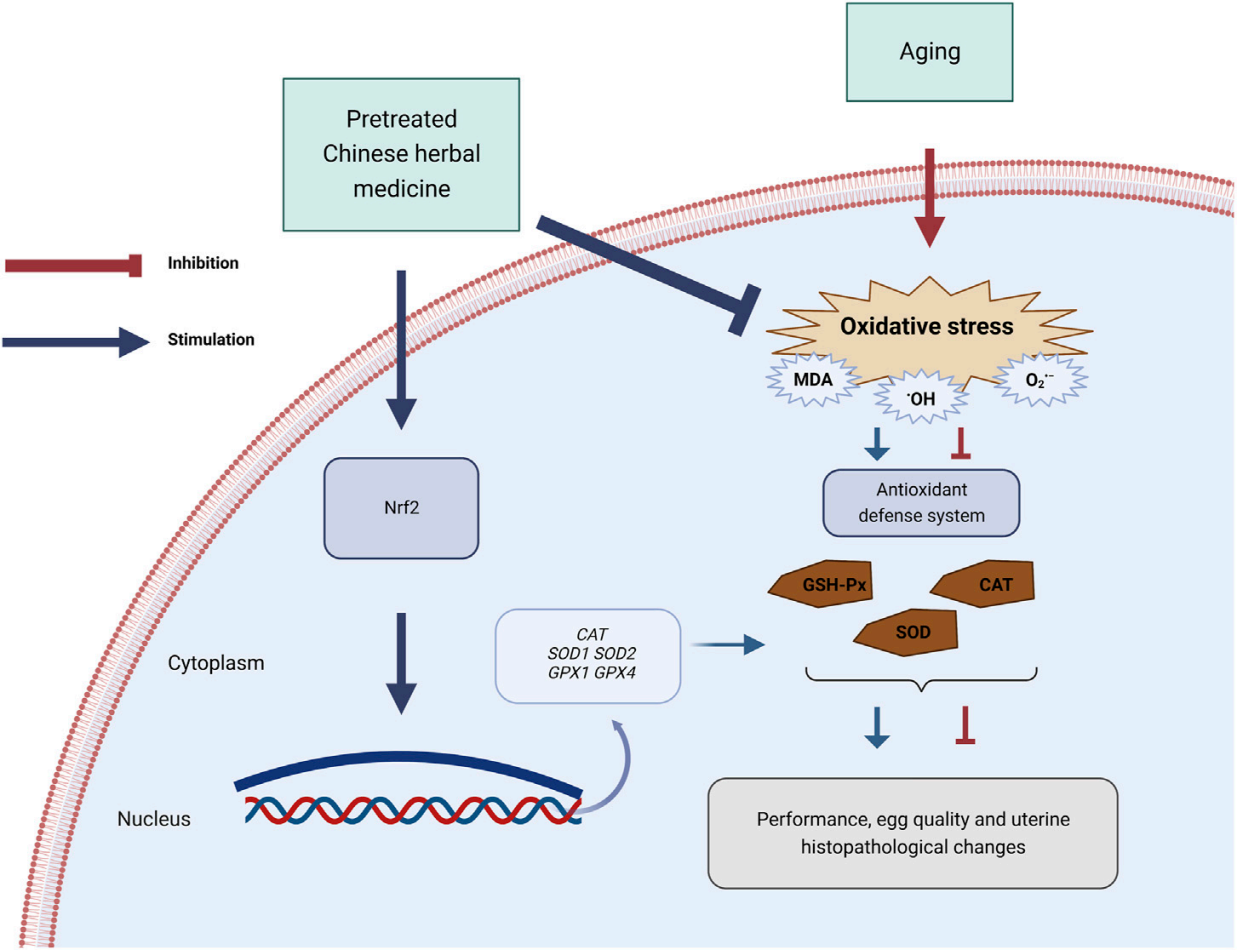
Schematic diagram summarizing the mechanisms by which pretreated Chinese herbal medicine (PCHM) promote the antioxidant defense system in late laying hens. The antioxidant defense system is downregulated in the natural aging process in laying hens. PCHM attenuated the oxidative stress in the uterus *via* the activation of the nuclear factor-erythroid 2-related factor 2 (*Nrf2*) pathway to increase the age-related up-produce in commercial laying hens. CAT, catalase; GSH-Px, glutathione peroxidase; SOD, superoxide dismutase; MDA, malondialdehyde; *SOD1*, Cu-Zn superoxide dismutase; *SOD2*, Mn superoxide dismutase; *CAT*, catalase; *GPX1*, glutathione peroxidase 1; *GPX4*, peroxidase 4. Figures generated with BioRender (https://biorender.com/).

In conclusion, PCHM supplementation boosted egg weight and quality in late-stage laying hens. This result may be attributable to the increase in antioxidant enzyme activity, which may be associated with increased antioxidant enzyme mRNA expression and *Nrf2* expression (Figure 9). However, the underlying mechanism through which dietary supplementation of PCHM enhances the mRNA expression of antioxidant enzymes requires additional investigation.

## Data Availability

The original contributions presented in the study are included in the article/supplementary materials, further inquiries can be directed to the corresponding authors.
